# AI-Driven sleep staging from actigraphy and heart rate

**DOI:** 10.1371/journal.pone.0285703

**Published:** 2023-05-17

**Authors:** Tzu-An Song, Samadrita Roy Chowdhury, Masoud Malekzadeh, Stephanie Harrison, Terri Blackwell Hoge, Susan Redline, Katie L. Stone, Richa Saxena, Shaun M. Purcell, Joyita Dutta

**Affiliations:** 1 University of Massachusetts Amherst, Amherst, MA, United States of America; 2 Massachusetts General Hospital, Boston, MA, United States of America; 3 Brigham and Women’s Hospital, Boston, MA, United States of America; 4 California Pacific Medical Center Research Institute, San Francisco, CA, United States of America; Vellore Institute of Technology: VIT University, INDIA

## Abstract

Sleep is an important indicator of a person’s health, and its accurate and cost-effective quantification is of great value in healthcare. The gold standard for sleep assessment and the clinical diagnosis of sleep disorders is polysomnography (PSG). However, PSG requires an overnight clinic visit and trained technicians to score the obtained multimodality data. Wrist-worn consumer devices, such as smartwatches, are a promising alternative to PSG because of their small form factor, continuous monitoring capability, and popularity. Unlike PSG, however, wearables-derived data are noisier and far less information-rich because of the fewer number of modalities and less accurate measurements due to their small form factor. Given these challenges, most consumer devices perform two-stage (i.e., sleep-wake) classification, which is inadequate for deep insights into a person’s sleep health. The challenging multi-class (three, four, or five-class) staging of sleep using data from wrist-worn wearables remains unresolved. The difference in the data quality between consumer-grade wearables and lab-grade clinical equipment is the motivation behind this study. In this paper, we present an artificial intelligence (AI) technique termed sequence-to-sequence LSTM for automated mobile sleep staging (SLAMSS), which can perform three-class (wake, NREM, REM) and four-class (wake, light, deep, REM) sleep classification from activity (i.e., wrist-accelerometry-derived locomotion) and two coarse heart rate measures—both of which can be reliably obtained from a consumer-grade wrist-wearable device. Our method relies on raw time-series datasets and obviates the need for manual feature selection. We validated our model using actigraphy and coarse heart rate data from two independent study populations: the Multi-Ethnic Study of Atherosclerosis (MESA; N = 808) cohort and the Osteoporotic Fractures in Men (MrOS; N = 817) cohort. SLAMSS achieves an overall accuracy of 79%, weighted F1 score of 0.80, 77% sensitivity, and 89% specificity for three-class sleep staging and an overall accuracy of 70-72%, weighted F1 score of 0.72-0.73, 64-66% sensitivity, and 89-90% specificity for four-class sleep staging in the MESA cohort. It yielded an overall accuracy of 77%, weighted F1 score of 0.77, 74% sensitivity, and 88% specificity for three-class sleep staging and an overall accuracy of 68-69%, weighted F1 score of 0.68-0.69, 60-63% sensitivity, and 88-89% specificity for four-class sleep staging in the MrOS cohort. These results were achieved with feature-poor inputs with a low temporal resolution. In addition, we extended our three-class staging model to an unrelated Apple Watch dataset. Importantly, SLAMSS predicts the duration of each sleep stage with high accuracy. This is especially significant for four-class sleep staging, where deep sleep is severely underrepresented. We show that, by appropriately choosing the loss function to address the inherent class imbalance, our method can accurately estimate deep sleep time (SLAMSS/MESA: 0.61±0.69 hours, PSG/MESA ground truth: 0.60±0.60 hours; SLAMSS/MrOS: 0.53±0.66 hours, PSG/MrOS ground truth: 0.55±0.57 hours;). Deep sleep quality and quantity are vital metrics and early indicators for a number of diseases. Our method, which enables accurate deep sleep estimation from wearables-derived data, is therefore promising for a variety of clinical applications requiring long-term deep sleep monitoring.

## Introduction

The quantity and quality of sleep are key indicators of human health [1]. Sleep is known to restore energy [2], bolster the immune system [3], ward off infection [4], and impact cognition and behavior [5]. Chronic disruptions in sleep patterns have been linked to cardiovascular disease [6], diabetes [7, 8], Alzheimer’s disease [9–11], depression [12], migraine [13], and many other serious conditions. Monitoring of sleep and the subsequent analysis and characterization of sleep patterns are, therefore, of long-standing interest to healthcare providers [14].

The gold standard for clinical sleep monitoring is overnight polysomnography (PSG), which is a multiparametric assessment of sleep featuring several functional measurements, including electroencephalography (EEG), electrocardiography (ECG), pulse oximetry, electrooculography (EOG), electromyography (EMG), and respiratory tracking (measurement of nasal pressure, thoracic effort, and abdominal effort). Following a PSG, trained technicians perform sleep scoring, which involves categorization of each 30-s sleep epoch into one of five sleep stages, namely, wake (W), rapid eye movement (REM) sleep, and three distinct categories of non-REM (NREM) sleep: N1, N2, and N3, as recognized by the American Academy of Sleep Medicine (AASM) [15]. Despite its universality in the clinic, PSG has serious limitations: It requires bulky equipment, is usually conducted in a clinical sleep laboratory by specialized personnel, and is unsuitable for continuous and long-term sleep monitoring. Manual sleep scoring following a PSG is a labor-intensive and expensive process that is limited by inter-rater scoring variability [16]. The development of reliable alternatives to this setup, encompassing both hardware (e.g., wearable devices for sleep monitoring) and software (automated techniques for sleep staging), remains an open research problem of high significance in sleep medicine and digital health [17–19].

Consumer-grade wearable devices are capable of continuous monitoring of sleep with the advantages of scalability and low cost over PSG. AASM has approved several specialized actigraphy devices for sleep vs. wake categorization (two-class sleep staging) [20–23]. In fact, two-class sleep staging is a well-established consumer technology beyond the clinic and a standard feature in many consumer-grade wrist wearables, e.g., smartwatches [24, 25]. In comparison, automated multi-class (i.e., three-, four-, or five-class) sleep staging from wearables-derived data remains an active area of research. Three-class staging involves categorizing each epoch as wake, REM, or NREM, while four-class staging further splits NREM into light (N1 and N2) and deep/slow-wave (N3) sleep categories. Methods for automated sleep staging have traditionally focused on EEG [26–29]. While portable EEG headbands exist, these are far from being universally adopted as consumer electronics. Recently, there has been significant enthusiasm surrounding ECG-based sleep scoring [30–33]. While wrist-based consumer smartwatch devices like the Apple Watch are capable of performing a single-lead ECG, this data cannot be acquired passively (e.g., when the wearer is asleep) or in real time as it requires conscious action on the wearer’s part (i.e., placing one’s finger on the sensor). Unlike ECG, heart rate is passively and continuously measured in these devices using photoplethysmography (PPG). As opposed to EEG- and ECG-based approaches which have limited on-the-go utility, an automated sleep staging technique that accepts activity and heart rate as the only two inputs will have broad applicability across a wide range of consumer-grade wrist-based devices and can be easily scaled to the fast-growing pool of smartwatch users.

It should be noted that four-class sleep staging is a more challenging machine learning problem than three-class staging because the four sleep classes are inherently severely imbalanced, i.e., there are major differences in the occurrence of the classes in a typical night. Specifically, among the three NREM sleep stages, N2 is the majority class and constitutes 45–55% of the total sleep period, while deep sleep (N3) comprises only about 10–20% [34]. This class imbalance problem curtails the performance of sleep staging algorithms and frequently leads to under-/over-estimation of individual sleep stages as previously noted [35–37]. Methods addressing class imbalance in machine learning are diverse and include the use of specialized loss functions such as class-balanced loss [38], cost-sensitive learning [39], or focal loss [40] and various forms of data augmentation such as synthetic minority oversampling (SMOTE) [41] and adaptive minority oversampling [42]. Such traditional oversampling methods, however, are not well-suited for sleep staging as the temporal correlations of the input time series must be maintained during the synthesis phase. The underrepresentation of deep sleep epochs makes four-class staging even more challenging when working with information-poor data from smartwatches and other wrist wearables compared to lab-grade ECG and EEG.

With the emergence of artificial intelligence (AI), new approaches for automated sleep scoring have become available. Unlike conventional approaches for EEG and ECG classification that are heavily reliant on feature handcrafting, deep learning techniques, such as convolutional neural networks (CNNs) and long short-term memory (LSTM) neural networks, can learn features that are the most discriminative for the sleep staging task directly from the raw data. In this paper, we present an AI technique that we coin sequence-to-sequence LSTM for automated mobile sleep staging (SLAMSS), which can perform three-stage and four-stage sleep classification using activity and two coarse heart rate measures (heart rate mean and heart rate standard deviation). The input data types for SLAMSS are compliant with a wide range of wrist wearables, which confers broad utility to our approach. The dependence on raw data obviates the need for manual feature selection. For performance evaluation, instead of relying solely on overall classification accuracy (which is often skewed in favor of the high-performing majority class when there is class imbalance), our approach emphasizes the accurate estimation of the duration of each sleep stage. A key focus of this work is the accurate computation of NREM sleep time, particularly the time spent in the N3 (or slow-wave/deep sleep) stage, as reduced N3 sleep is implicated in a broad range of serious disorders. It should be noted that the N3 staging is challenging and typically relies on EEG data—even efforts based on lab-grade ECG have led to significant under- or overestimation of the N3 stage [32].

To train and test the SLAMSS network, we use activity and heart rate measures derived from the Multi-Ethnic Study of Atherosclerosis (MESA) dataset [43, 44]. To show the broad utility of our model, we also validate the model using another independent dataset, the Osteoporotic Fractures in Men (MrOS) cohort [43, 45]. The demographics of the two datasets are summarized in Table 1. Activity measures from both MESA and MrOS are based on wrist actigraphy, albeit based on different devices. MESA activity measures are available at 30-s intervals, whereas MrOS activity measures were available at 60-s intervals. For mobile-grade cardiac measures, we derived two coarse metrics from MESA and MrOS ECG: heart rate mean and heart rate standard deviation over 30-s epochs. We use manual sleep scores from multi-site PSG (but from a single central Sleep Reading Center for each cohort) as the ground truth for model training and validation. In order to show the wide utility of the model, we also test the model on an independent mobile dataset based on the Apple Watch [46]. Finally, we address the significant challenge that class imbalance poses to N3 sleep staging through the introduction of “real-world weighting” in the loss function that is minimized during the model training phase. To the best of our knowledge, SLAMSS is the first example of an AI technique that is able to work for three- and four-class sleep staging from wearables-grade inputs and provides clinically relevant, accurate estimates of sleep-stage durations. What distinguishes our method is its ability to achieve good performance using coarse inputs with a low temporal resolution.

**Table 1:** MESA and MrOS participant demographics in this study.

Dataset		MESA	MrOS
Number of subjects		808	817
Age [mean(s.d.)]		69.27 (9.00)	72.93 (5.41)
Sex	Male	373	817
	Female	435	–
Race	White Caucasian	327	762
	Chinese American	82	18
	Black African American	229	19
	Hispanic	170	8
	Other	–	10

## Materials and methods

### MESA dataset

This paper relies on the secondary use of publicly available de-identified data from the MESA Sleep Study [43, 44]. MESA is a multi-center longitudinal investigation designed to research the transition of sub-clinical to clinical cardiovascular disease [47]. The study comprises 6,814 asymptomatic men and women of black, white, Hispanic, and Chinese-American ethnicity, of which 2,237 were also enrolled in the MESA Sleep Study. Participants of the sleep study wore an actigraphy device for one week. They also underwent one full night of PSG while wearing the actigraphy device, thus providing synchronous actigraphy and PSG data. Data analysis for this study only included concurrently acquired actigraphy and PSG data. MESA sleep data were collected from six field centers across the United States: Wake Forest University, Columbia University, Johns Hopkins University, University of Minnesota, Northwestern University, and University of California Los Angeles. MESA protocols were approved by the Institutional Review Board at each field center, and all participants gave written informed consent as described in prior publications [43, 44].

#### Actigraphic inputs

MESA actigraphy data were collected using the Actiwatch Spectrum device from Philips Respironics. The device, fastened to a participant’s wrist, captures triaxial acceleration signals and converts them to activity counts reflecting subjective upper limb locomotion. An aggregate activity count for each 30-s epoch is available for synchronous comparison with PSG.

#### Cardiac inputs

Cardiac sensing is unavailable from the Actiwatch actigraphy device used in the MESA study. We extracted a coarse-grained measure of heart rate from the ECG (with bipolar leads) acquired as part of the PSG. The heart rate is derived from the consecutive R point locations available for each subject. The reciprocal of the gap between two consecutive R points is the instantaneous heart rate (IHR). We averaged the IHR over each 30-s epoch to arrive at the heart rate mean (HRM) time series. The standard deviation of the IHR over each 30-s epoch generates the heart rate standard deviation (HRSD) time series, which can be thought of as a simple heart rate variability (HRV) measure. These two coarse measures were chosen as they can be easily derived from PPG heart rate data available in real time from most consumer smartwatch devices and do not require an ECG.

#### PSG scoring

PSG was performed using a Compumedics Somte Sleep Monitoring System. The sensors of the Compumedics PSG device used in the MESA study acquire multimodal data in the form of bio-electrical potentials (EEG, EOG, EMG, and ECG), pressure measures (respiratory bands), and oximetry [44]. The data were scored following AASM guidelines by trained technicians with high levels of inter- and intra-scorer reliability with inter-class correlation coefficients typically exceeding 0.85. Sleep scoring for the entire cohort was performed at a central Sleep Reading Center. The PSG-based epoch-by-epoch sleep labels included in the MESA dataset served as our ground truth for model training and testing.

#### Data preprocessing

For the purpose of this study, 808 subjects were selected among the 2, 237 participants based on the quality of the PSG and the availability of concurrent PSG and actigraphy data. MESA provides indicators of PSG and actigraphy data quality determined by the duration of artifact-free data across channels, with 1 being the worst quality score and 7 the best quality score. To ensure data integrity and sleep hypnogram precision, we used data from all participants with a PSG quality rating ≥ 6 and for whom there is concurrent actigraphy and PSG data with PSG-actigraphy overlap duration ≥ 6.6 hours. We rectified any misalignment between actigraphy and cardiac measures in the dataset using the cross-correlation between the activity and heart rate time series with MATLAB’s cross-correlation function (xcorr).

In MESA, R-points were detected using the Compumedics (Abbotsford, VIC, Australia) Somte software Version 2.10. To obtain the IHR from the R-peaks, we first extracted the time interval between consecutive R-peaks for each epoch. For any interval that was below 0.33 s (which can result in a heart rate above 180 bpm during the night), we replaced it with the midpoint of that interval and the previous interval. In contrast, for intervals longer than 1.33 s, which could result in a heart rate of 46 bpm or lower (a rare occurrence), we uniformly divided the space between the current and previous interval into *T*/*T*_*mean*_ chunks, where *T* is the current interval and *T*_*mean*_ is the mean interval for that epoch. Next, we computed the mean and standard deviation of the extracted heart rate values for each epoch. Any heart rate value that was outside of two standard deviations from the mean was discarded to eliminate outliers.

### MrOS dataset

For independent validation in a second cohort, we rely on the secondary use of publicly available de-identified data from the MrOS Sleep Study [43, 45]. MrOS, a study originally designed to determine the epidemiology of osteoporotic fractures in men, has greatly broadened in its scope over the years. This multi-site study features data from 5,994 community-dwelling men 65 years or older. The MrOS Sleep Study is an ancillary study in which actigraphy and/or PSG data were collected from a subset of the cohort (*N* = 3058). 896 men in this sub-cohort had concurrent PSG and actigraphy recordings, of which *N* = 817 participants had a PSG-actigraphy overlap duration ≥ 2 hours and were, therefore used in our analysis. MrOS sleep data were collected across six clinical sites: University of Alabama at Birmingham, Oregon Health and Science University, Stanford University, University of Minnesota, University of Pittsburgh, and University of California San Diego. Institutional Review Board approvals and written informed consent were obtained from all participants at each of the six sites as described in prior publications [43, 45].

#### Actigraphic inputs

MrOS actigraphy data were collected using the Sleepwatch-O actigraph (Ambulatory Monitoring, Inc, Ardsley, NY). This is a wrist-worn device which captures and aggregates motion signals over 60-s epochs. The temporal resolution of MrOS actigraphy measures is, therefore, lower than that for MESA, which reports activities at 30-s intervals. Because PSG-derived sleep labels and HRM and HRSD measures are available for each 30-s epoch, the activity counts were upsampled to match the 30-s temporal resolution of the remaining time series by replicating the activity used for each 60-s epoch into two 30-s epochs.

#### Cardiac inputs

We extracted coarse-grained HRM and HRSD measures at 30-epoch intervals from the ECG acquired as part of the PSG.

#### PSG scoring

PSG was performed using a Compumedics Safiro Sleep Monitoring System. Sleep staging was performed in 30-s scoring epochs by scorers, who were blinded to the sleep-wake status from the actigraphy device. It should be noted that PSG scoring for MrOS was performed at the same central Sleep Reading Center used for the MESA cohort.

#### Data preprocessing

The 817 subjects used in our study constitute the subset of MrOS participants with concurrent actigraphy and PSG data with PSG-actigraphy overlap duration > 2 hours. A time-series cross-correlation metric was used to rectify any misalignment between actigraphy and cardiac measures.

### Apple Watch dataset

This paper relies on the secondary use of publicly available de-identified data from an Apple Watch study conducted at the University of Michigan [46]. The Apple Watch dataset was collected from 2017 to 2019 with approval by the University of Michigan Institutional Review Board and made publicly available via PhysioNet [48, 49]. This cohort consists of 31 subjects (21 female) with ages ranging from 19 to 55 years. The subjects wore an Apple Watch (Series 2 or 3) to acquire PPG-derived heart rate and raw accelerometry data simultaneously with a PSG recording. The PSGs were scored by professional technicians following AASM guidelines.

The raw accelerometer data from the Apple Watch has *x*, *y*, and *z* components that correspond to different wrist movement directions, with the *z*-component being the most sensitive to the activity [50]. To convert raw data into counts, only the *z*-component of the accelerometry data was used, and a band-pass filter was applied to remove the gravitational rotation signal. The signal was then divided into 128 bins and summed over 15-s windows to obtain one count value per 15-s epoch.

### Overview of SLAMSS

**Fig 1:**
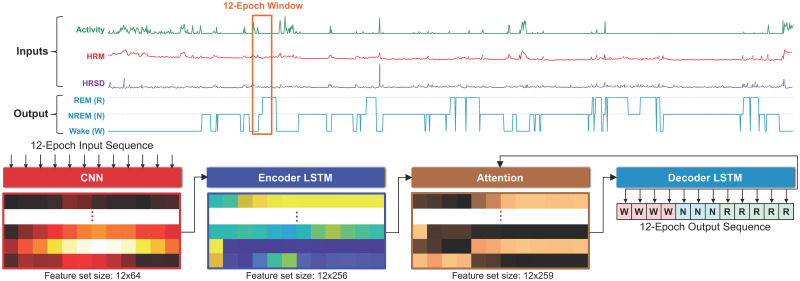
Network overview. SLAMSS network architecture showing sample activity, heart rate mean (HRM) and heart rate standard deviation (HRSD) time series that are the model inputs and sleep stage labels Wake (W), REM (R), and NREM (N) that are the model outputs. Our SLAMSS network implementation operates on 12-epoch input sequences that are passed into a set of CNNs (epoch duration = 30 s). The CNN features go through an attention-guided encoder-decoder system that generates the output labels.

The SLAMSS network receives as inputs activity, HRM, and HRSD time series provided as fixed-length sequences of epochs as shown in Fig 1 and generates as outputs sleep stage labels (wake, REM, and NREM for three-stage classification and wake, light, deep, and REM for four stage classification). The network uses a sequence-to-sequence (Seq2Seq) architecture [51] combined with a CNN. The CNN layers extract linear convolutional features and pass them through a nonlinear activation function. CNN-derived features are fed into the Seq2Seq model, which consists of an encoder and a decoder, each composed of LSTM units that capture the long short-term contextual correlations between inputs and targets. A hidden state generated by the encoder is fed to the decoder through an attention layer. Intuitively, the attention mechanism creates an attention vector by combining features from all epochs that have been witnessed by the network at a given point of the sequential processing setup. The decoder output passes through a softmax layer, which computes the probability of a particular epoch belonging to a given sleep stage. The model is trained in a supervised manner with the PSG sleep stage labels for each epoch as the target.

### Network architecture

The SLAMSS classifier utilizes a Seq2Seq architecture consisting of an LSTM combined with a CNN. A rough schematic of the network architecture is shown in Fig 1. The CNN comprises three consecutive one-dimensional convolutional layers, with a kernel size of 9, padding 4, and stride 1 followed by leaky rectified linear units (leaky ReLUs). The generated features are subsequently utilized as inputs for the Seq2Seq model, which employs an encoder-decoder structure equipped with attention. In the Seq2Seq model, both the encoder and decoder are composed of LSTM units that can extract and capture long-term and short-term contextual relationships between the input and target. The attention mechanism constitutes a hidden representation created by the encoder, which integrates features from all epochs. This hidden state undergoes an attention-focused optimization process [52]. This method generates an alignment score by comparing the encoder’s hidden state with the decoder’s hidden state. Attention weights are determined by applying a softmax activation function to these alignment scores. Finally, a context vector, which is a weighted sum of the attention weights and encoder hidden states, is generated. Attention-based optimization ensures that the model learns the most relevant parts of an incoming sequence. It prevents the model from overemphasizing the last element of the sequence as is often the case with non-attention-driven decoders [52]. SLAMSS employs a 12-epoch (6-min) long sliding window with the stride set to 1 epoch. In other words, a given 12-epoch input sequence has 11 of its epochs overlapping with its predecessor. As a result of this overlap, every epoch is labeled 12 times by the classifier except for the first 11 and the last 11 epochs in the full time series (i.e., the data from one participant in one full night/day). The final output label is determined by computing the mode (i.e., the most frequently occurring value) of the 12 assigned labels.

### Network training

The network was implemented and trained on PyTorch using an NVIDIA GTX 2080 graphics card. All data were divided subject-wise into independent training, validation, and test datasets. The hyperparameters learning rate and batch size were set at 0.00015 and 50 respectively. Using the Adam optimizer, the model was trained for 200 optimization epochs. The validation dataset was used to identify the best set of model parameters. These model parameters were then used to generate our final reported results from the test dataset. Different loss functions were used for different classification tasks, as explained below.

#### Loss function for three-class sleep staging

The categorical cross-entropy loss is a well-accepted loss function for multi-class classification. Three-class sleep staging trisects sleep data into wake, NREM, and REM stages. The three classes are mildly imbalanced. To address this imbalance, we weight the standard cross-entropy loss by the inverse of the frequency for each stage. The inverse frequency (IF) weighted loss, denoted below as *J*_IF_, is given by:

$$\begin{array}{c} J_{\text{IF}}=-\frac{1}{M}\sum_{m=1}^{M}\sum_{k=1}^{K}w^{k}y_{m}^{k}\;\text{log}\left(h_{\theta }^{k}\left(x_{m}\right)\right), \end{array}$$ (1)

where *M* is the number of training examples, *K* is the number of classes, $y_{m}^{k}$ is the target label for the *m*th training sample belonging to class *k*, *x*_*m*_ is the input for the *m*th training sample, *w*^*k*^ is the IF weight corresponding to the *k*th class, *h*_*θ*_ refers to the parametric (neural network) model, and *θ* is the set of model parameters (i.e., neural network weights).

#### Loss function for four-class sleep staging

Four-class sleep staging assigns each epoch of sleep into wake, light (N1+N2), deep (N3), and REM stages. This classification task requires more aggressive mitigation of class imbalance due to the severe underrepresentation of the deep sleep stage. Though IF weighting helps address class imbalance, for the four-class sleep staging task, it increases minority class accuracy/sensitivity at the expense of erroneous overestimation of the minority class. The latter could lead to a gross overestimation of deep sleep time and deep sleep fraction. To avoid deep sleep overestimation, it is essential that the classifier can correctly identify the true positives without increasing the number of false positives. To accomplish that, we adopted a novel weighting scheme for the loss function referred to as real-world (RW) weighting [53], which weights the cross-entropy loss function in a way so as to penalize both the actions of missing a positive and misidentifying a negative as a positive. The RW-weighted function denoted below as *J*_RW_, is formulated as:

$$\begin{array}{c} J_{\text{RW}}=-\frac{1}{M}\sum_{m=1}^{M}[\sum_{k=1}^{K}w_{\text{fn}}^{k}y_{m}^{k}\;\text{log}\left(h_{\theta }^{k}\left(x_{m}\right)\right)+\sum_{k=1}^{K}\sum_{\begin{array}{c} l=1\\ l\neq k \end{array}}^{K}w_{\text{fp}}^{kl}\;y_{m}^{k}\;\text{log}\left(1-h_{\theta }^{l}\left(x_{m}\right)\right)], \end{array}$$ (2)

where *M* is the number of training examples, *K* is the number of classes, $y_{m}^{k}$ is the target label for the *m*th training sample belonging to class *k*, *x*_*m*_ is the input for the *m*th training sample, $w_{\text{fn}}^{k}$ is the marginal cost of a false negative over a true positive, $w_{\text{fp}}^{kl}$ is the marginal cost of a false positive of class *l* over a true negative, when the true positive is class *k*, *h*_*θ*_ refers to the parametric (neural network) model, and *θ* is the set of model parameters (i.e., neural network weights).

The parameters $w_{\text{fp}}^{kl}$ can be thought of as the elements of a matrix with zeros for the diagonal elements and with the off-diagonal terms expressing the penalty imposed on the model for generating false positives. This set of parameters is determined and defined heuristically based on the underlying constraints of the data distribution in a given dataset. The idea is that a network trained using this weight matrix may use the same rules of thumb to predict other datasets. To populate these elements, we used the square root of the relevant pair of inverse frequencies of the sleep stages from the training dataset, as shown in S1 Fig.

### Evaluation metrics

To assess the performance of our SLAMSS classifier, we use a set of model performance metrics commonly accepted in the machine learning community as well as a set of clinical sleep metrics. The most detailed indicator of a model’s performance is the confusion matrix. The diagonal elements of this matrix capture the accuracy of the model. For three-class sleep staging, the confusion matrix captures the model’s effectiveness at trisecting the dataset into the categories wake, NREM sleep, and REM sleep. The diagonal and off-diagonal elements respectively represent the proportions of correct and incorrect classification. Apart from sensitivity and specificity, we compute metrics that simultaneously account for precision and recall for the algorithm. The first of these is the *F*_1_ score, which is the harmonic mean of precision and recall. The efficacy of the *F*_1_ score is constrained by the fact that it ignores true negatives. This leads us to compute the Matthews Correlation Coefficient (MCC), the only metric that simultaneously captures all the entries in the confusion matrix to create a single scalar measure. The MCC computes the correlation between observed and predicted binary classes and thus is symmetric to positive and negative class definitions. In our multi-class scenario, we calculate the MCC for each class, as “one vs. the rest” and provide the macro-averaged MCC as a single representative number.

The above-mentioned metrics provide a quantitative assessment of the performance of the classifier model. However, the ultimate clinical relevance of a sleep staging model is determined by its accuracy at computing sleep metrics that rely on the stage label assignment. To assess the clinical impact of our method, we, therefore, calculate a series of sleep metrics that are well-defined in the literature, e.g., sleep efficiency, sleep onset latency, sleep fragmentation, and total sleep time in hours. We also report the fractional time spent in each sleep stage. In addition, we have computed a sleep metric termed *sleep transition index* [23, 54–56]. This index measures the time spent in transitions after sleep onset over the course of the night normalized by the time spent sleeping after the sleep latency period. Probabilistic rates of transition between sleep stages have been used as markers of sleep continuity for the purpose of sleep quality assessment [54]. Higher probabilities of sleep transitions are known to be associated with disorders such as chronic fatigue syndrome [57]. The transition metric, that we define here has value in enumerating the shifts in sleep stages and could be used as an indicator of sleep organization. The definitions of all evaluation metrics are provided in S1 and S2 Tables.

## Results

### Three-class sleep staging using the MESA dataset

**Fig 2:**
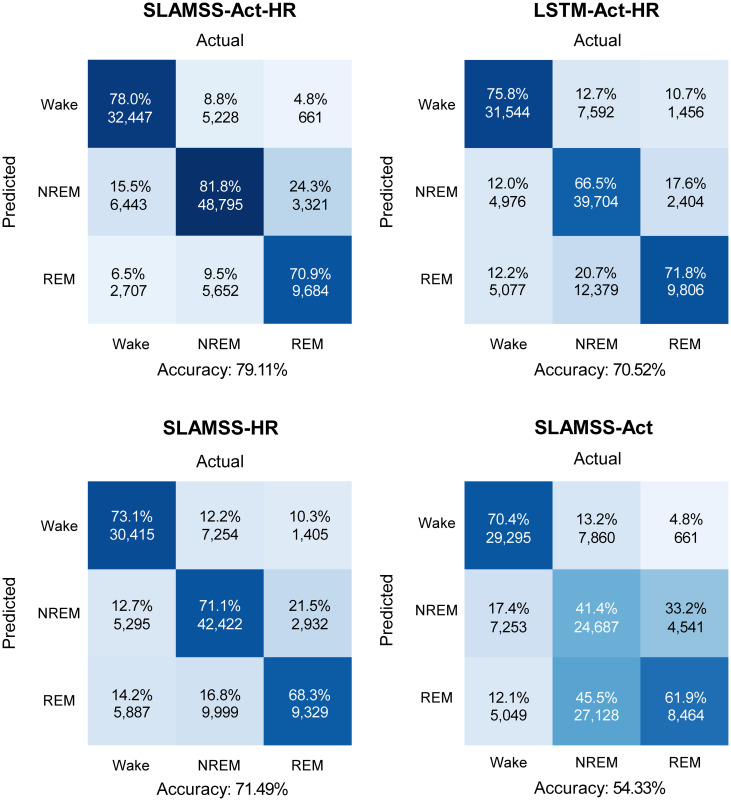
MESA three-class sleep staging. Confusion matrices for four classifiers: SLAMSS with activity, HRM, and HRSD inputs (SLAMSS-Act-HR), LSTM with activity, HRM, and HRSD inputs (LSTM-Act-HR), SLAMSS with HRM and HRSD inputs (SLAMSS-HR), and SLAMSS with an activity input (SLAMSS-Act). Each box shows % epochs at the top and the number of epochs below. Columns sum to 100%. Expert manual sleep staging by PSG is used as the ground truth. It should be noted that, for three-class staging, category assignment by random chance would lead to a value of 33.33% for the diagonal elements of these matrices.

We trained and tested the SLAMSS network on data from *N* = 808 MESA participants who had concurrent PSG and actigraphy. 608 participants (75%) were randomly assigned to a training subset, 100 (12.5%) to a validation subset, and the remaining 100 (12.5%) to an independent test subset. Network parameters were computed in the training phase by minimizing a cross-entropy loss function with inverse frequency weighting for each sleep stage. We compared the performance of the SLAMSS architecture for three-class sleep staging with that of a conventional LSTM. Fig 2 shows the three-class confusion matrices for the SLAMSS and LSTM models receiving the full set of inputs, i.e., activity, HRM, and HRSD (abbreviated as SLAMSS-Act-HR and LSTM-Act-HR, respectively). SLAMSS-Act-HR correctly identifies 78.0% of wake epochs, 81.8% of NREM epochs, and 70.9% of REM epochs, with a notable accuracy margin of 15.3% over LSTM-Act-HR for NREM and smaller improvements for wake. We have also examined the performance of SLAMSS with HRM and HRSD as inputs (SLAMSS-HR) and SLAMSS with activity as the only input (SLAMSS-Act). While SLAMSS-HR is more accurate for all classes than SLAMSS-Act (which, as expected, yields low accuracy overall, with the lowest number being that for NREM sleep), the combination of activity and heart rate inputs simultaneously boosts accuracy for all three classes. In addition, we also trained SLAMSS-Act-HR with various input window widths, and the comparison result is shown in S2 Fig.

For reference, we have included the accuracies reported in other papers for three- and four-class sleep staging on activity and cardiac data in Table 2. This table excludes methods that are dependent on clinical-grade ECG [32] or an extensive array of hand-crafted ECG features [31, 58]. In principle, the SLAMSS network performance could substantially improve with the introduction of information-rich, handcrafted ECG features. However, we purposefully restrict ourselves to simple HRM and HRSD inputs as these can be reliably computed from a smartwatch PPG signal. We note that available consumer smartwatches, while capable of generating a single-lead ECG on demand, cannot acquire an ECG in the background in real time, e.g., when a subject is sleeping. It is noteworthy that Zhai et al. [59] reported severely diminished performance for the minority class (i.e., REM for three-class staging and deep sleep for four-class staging), a challenge that SLAMSS is able to overcome. SLAMSS also shows substantially better accuracies for all classes than Boe et al. [60], despite the fact that the latter utilizes real-time ECG and temperature signals that are typically not available through a consumer smartwatch device.

**Table 2:** Performance comparison of SLAMSS with other publications performing three or four-class sleep staging on activity (Act) and heart rate or heart rate variability (HRV) data.

Reference	Modalities	Needs real-time ECG for cardiac features?	Classifier	Nights	3-Class Sensitivity			4-Class Sensitivity			
					Wake	NREM	REM	Wake	Light	Deep	REM
This paper	Act, HRM, HRSD	No	SLAMSS	808	78.0%	81.8%	70.9%	78.7%	66.3%	55.9%	63.0%
Zhai et al., 2020	Act, 8 ECG features	Yes	CNN+LSTM	1, 743	75.0%	84.0%	42.0%	77.0%	80.0%	4.0%	55.0%
Walch et al., 2020	Act, HRM, HRV	No	Neural Network	31	60.0%	62.2%	62.5%	-	-	-	-
Boe et al., 2019	Act, ECG, Temperature	Yes	Bagging	11	73.3%	59.0%	56.0%	72.0%	56.0%	30.4%	31.6%

Additional metrics for assessing the classifier performance are listed in Table 3. SLAMSS-Act-HR outperforms LSTM-Act-HR for all five model performance metrics. SLAMSS-Act-HR not only outperforms the other models in the oft-cited sensitivity, specificity, and precision measures, but its considerable performance margins in the more balanced *F*_1_ score and MCC are indicative of the overall macro-level superiority of the model. To provide a clear effect size, we have also computed the Mean Absolute Error (MAE) for clinical metrics and the results are shown in Table 4. SLAMSS-Act-HR outperforms LSTM-Act-HR in all clinical sleep metrics except total sleep time. In addition, compared to LSTM-Act-HR, all SLAMSS variants exhibit lower error values for sleep onset latency and sleep transition index.

**Table 3:** MESA three-class sleep staging. Comparison of classifier performance metrics for four classifiers: SLAMSS with activity, HRM, and HRSD inputs (SLAMSS-Act-HR), LSTM with activity, HRM, and HRSD inputs (LSTM-Act-HR), SLAMSS with HRM and HRSD inputs (SLAMSS-HR), and SLAMSS with an activity input (SLAMSS-Act). PSG is used as the ground truth for the computation of all metrics. Subject-wise values are reported as mean(s.d.).

Metric	SLAMSS-Act-HR	LSTM-Act-HR	SLAMSS-HR	SLAMSS-Act
**Overall Accuracy**	0.79	0.71	0.71	0.56
**Sensitivity/Recall**	0.77	0.71	0.71	0.61
**Specificity**	0.89	0.86	0.86	0.81
**Precision**	0.74	0.66	0.66	0.59
**Weighted *F*_1_ score**	0.80	0.72	0.73	0.59
**Subject-wise**	0.80 (0.01)	0.72 (0.02)	0.73 (0.02)	0.59 (0.02)
**Weighted MCC**	0.66	0.56	0.57	0.44
**Subject-wise**	0.66 (0.02)	0.56 (0.03)	0.56 (0.03)	0.37 (0.02)

**Table 4:** MESA three-class sleep staging. Comparison of MAE for clinical sleep metrics for four classifiers against PSG: SLAMSS with activity, HRM, and HRSD inputs (SLAMSS-Act-HR), LSTM with activity, HRM, and HRSD inputs (LSTM-Act-HR), SLAMSS with HRM and HRSD inputs (SLAMSS-HR), and SLAMSS with an activity input (SLAMSS-Act). MAE values are provided in the format: mean (s.d.).

Metric	SLAMSS-Act-HR	LSTM-Act-HR	SLAMSS-HR	SLAMSS-Act
**Sleep efficiency**	0.08 (0.07)	0.07 (0.06)	0.09 (0.08)	0.10 (0.09)
**Sleep onset latency (min.)**	59.21 (94.06)	82.09 (105.29)	74.47 (99.49)	61.34 (100.42)
**Sleep fragmentation**	0.20 (0.44)	0.22 (0.24)	0.24 (0.39)	0.25 (0.35)
**Sleep transition index**	0.03 (0.02)	0.09 (0.05)	0.03 (0.03)	0.04 (0.03)
**NREM time (hrs.)**	0.73 (0.64)	1.18 (0.98)	1.30 (1.06)	1.99 (1.23)
**REM time (hrs.)**	0.55 (0.46)	1.19 (1.00)	1.05 (0.94)	2.02 (1.35)
**Total sleep time (hrs.)**	0.72 (0.70)	0.68 (0.62)	0.84 (0.74)	0.95 (0.99)
**NREM fraction**	0.08 (0.07)	0.19 (0.15)	0.16 (0.13)	0.31 (0.17)
**REM fraction**	0.08 (0.07)	0.19 (0.15)	0.16 (0.13)	0.31 (0.17)

**Fig 3:**
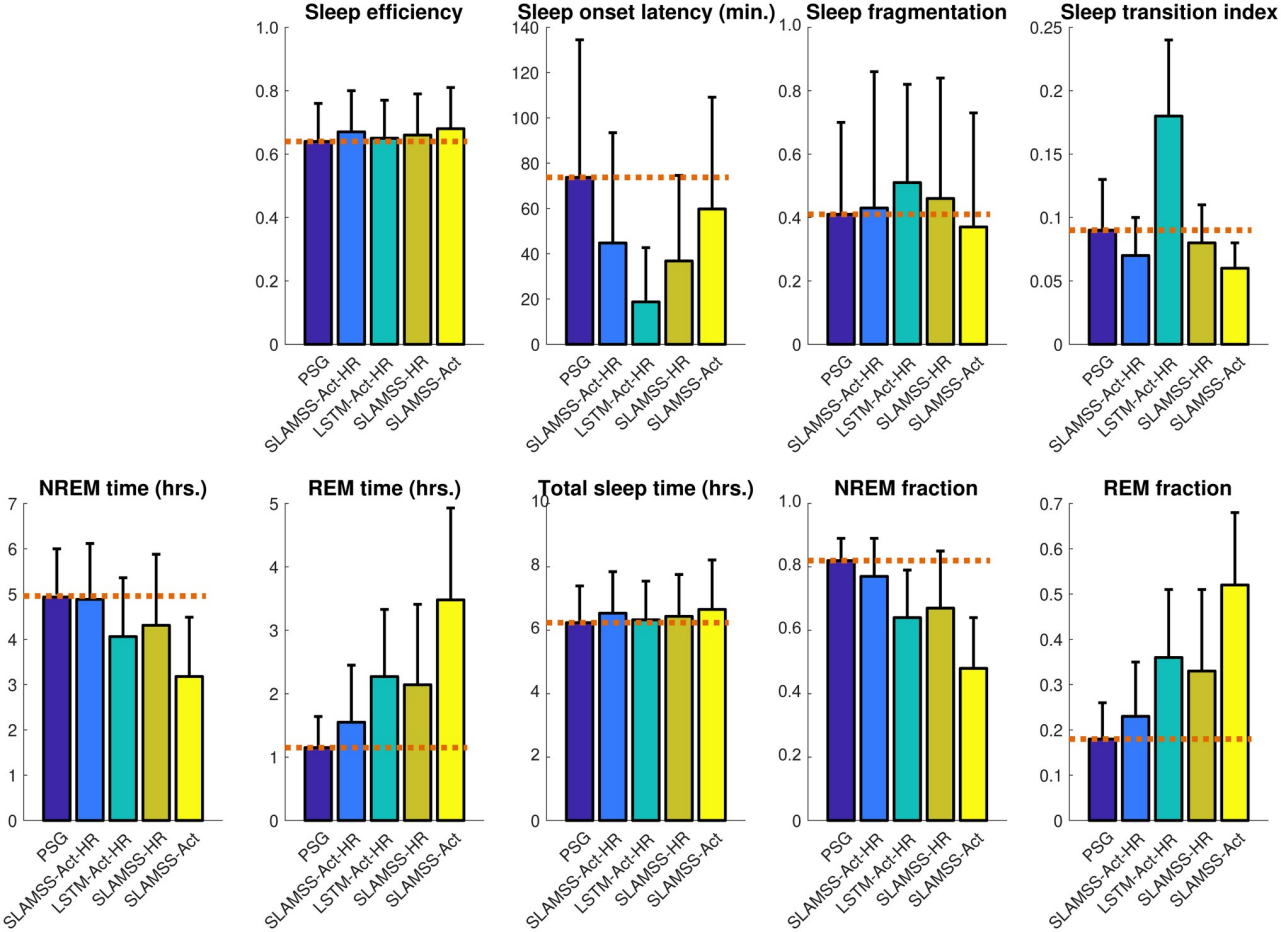
MESA three-class sleep staging. Comparison of clinical sleep metrics for four classifiers: SLAMSS with activity, HRM, and HRSD inputs (SLAMSS-Act-HR), LSTM with activity, HRM, and HRSD inputs (LSTM-Act-HR), SLAMSS with HRM and HRSD inputs (SLAMSS-HR), and SLAMSS with an activity input (SLAMSS-Act). The orange dotted line corresponds to the PSG (assumed ground truth) value of each metric.

Fig 3 showcases clinical sleep metrics computed for each classifier with the corresponding PSG-derived ground-truth numbers provided for reference. A key observation from this figure is that SLAMSS-Act-HR’s estimate of NREM sleep time (4.98 ± 1.16 hrs.) is especially close to that suggested by PSG (4.99 ± 1.05 hrs.). SLAMSS-Act-HR also produces REM sleep time estimates that are the closest to PSG. While LSTM-Act-HR generates a more accurate total sleep time measure and thereby could be sufficient for binary sleep-wake classification, it underestimates NREM time by around 20% and overestimates REM time by 101% on average. SLAMSS-Act-HR estimates sleep fragmentation more accurately than other approaches. All compared classifiers underestimated sleep onset latency. It must be noted here that the estimation of this metric using coarse actigraphic measures is notoriously challenging, and many previous papers have reported severe underestimation of this metric [61]. Our findings suggest that a coarse heart rate feature is even less effective than actigraphy alone, and the combination of activity and heart rate (i.e., SLAMSS-Act-HR) leads to intermediate accuracy for this metric. It must be noted here that, while many competing definitions of sleep onset latency exist in literature as reported in [62], we defined this metric here as the first detected period of three consecutive sleep epochs, which is the most stringent and popular definition. All versions of SLAMSS led to comparable estimates for the sleep transition index, with SLAMSS-HR producing the most accurate result. The measurement of sleep transitions is intricately linked to temporal correlations in the data [63]. The notable performance margin for this metric between the SLAMSS variants and LSTM suggests that SLAMSS is more effective at learning temporal correlations than LSTM.

### Three-class sleep staging using the MrOS dataset

**Fig 4:**
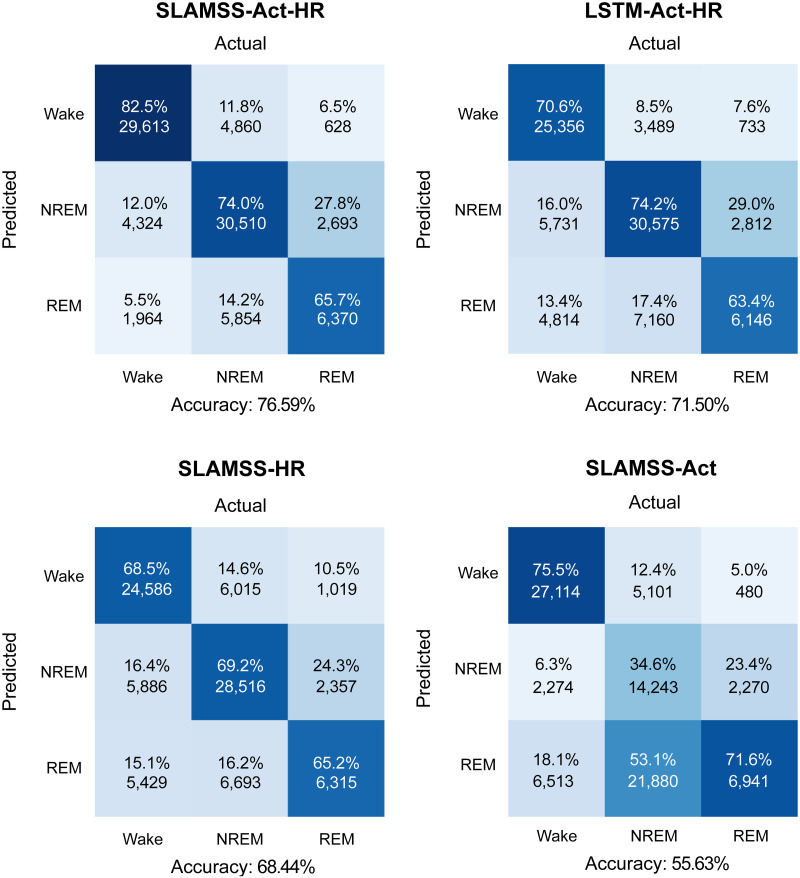
MrOS three-class sleep staging. Confusion matrices for four classifiers: SLAMSS with activity, HRM, and HRSD inputs (SLAMSS-Act-HR), LSTM with activity, HRM, and HRSD inputs (LSTM-Act-HR), SLAMSS with HRM and HRSD inputs (SLAMSS-HR), and SLAMSS with an activity input (SLAMSS-Act). Each box shows % epochs at the top and the number of epochs below. Columns sum to 100%. Expert manual sleep staging by PSG is used as the ground truth. It should be noted that, for three-class staging, category assignment by random chance would lead to a value of 33.33% for the diagonal elements of these matrices.

We trained and tested the SLAMSS network on data from *N* = 817 MrOS participants who had concurrent PSG and actigraphy. 617 participants (75%) were randomly assigned to a training subset, 100 (12.5%) to a validation subset, and the remaining 100 (12.5%) to an independent test subset. As with MESA three-class sleep staging, a cross-entropy loss function with IF weighting for each sleep stage was employed. A comparison of confusion matrices for three-class sleep staging in the MrOS cohort using SLAMSS-Act-HR, LSTM-Act-HR, SLAMSS-HR, and SLAMSS-Act is shown in Fig 4. The average accuracies of all methods are comparable across the MESA and MrOS cohorts. In the MrOS cohort, SLAMSS-Act-HR correctly identifies 82.5% of wake epochs, 74.0% of NREM epochs, and 65.7% of REM epochs. It has an 11.9% margin over LSTM-Act-HR at wake epoch identification. Additional metrics for assessing the classifier performance in the MrOS cohort are provided in Table 5. SLAMSS-Act-HR outperforms reference approaches in terms of all five model performance metrics.

**Table 5:** MrOS three-class sleep staging. Comparison of classifier performance metrics for four classifiers: SLAMSS with activity, HRM, and HRSD inputs (SLAMSS-Act-HR), LSTM with activity, HRM, and HRSD inputs (LSTM-Act-HR), SLAMSS with HRM and HRSD inputs (SLAMSS-HR), and SLAMSS with an activity input (SLAMSS-Act). PSG is used as the ground truth for the computation of all metrics. Subject-wise values are reported as mean(s.d.).

Metric	SLAMSS-Act-HR	LSTM-Act-HR	SLAMSS-HR	SLAMSS-Act
**Overall Accuracy**	0.77	0.72	0.68	0.56
**Sensitivity/Recall**	0.74	0.69	0.68	0.61
**Specificity**	0.88	0.86	0.84	0.81
**Precision**	0.70	0.66	0.63	0.59
**Weighted *F*_1_ score**	0.77	0.73	0.70	0.59
**Subject-wise**	0.77 (0.02)	0.73 (0.02)	0.71 (0.02)	0.58 (0.05)
**Weighted MCC**	0.63	0.57	0.52	0.44
**Subject-wise**	0.63 (0.03)	0.56 (0.03)	0.52 (0.03)	0.42 (0.04)

**Fig 5:**
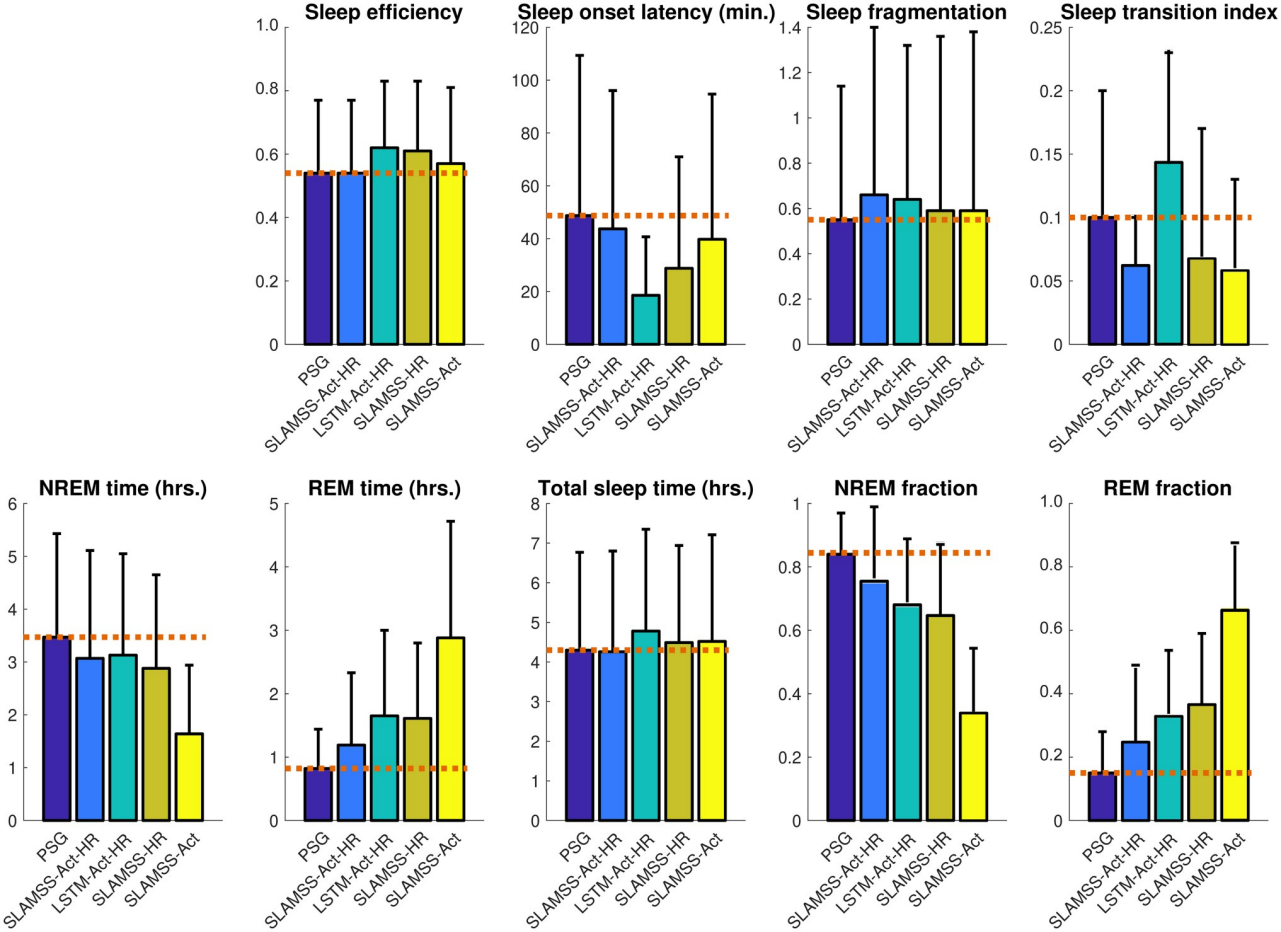
MrOS three-class sleep staging. Comparison of clinical sleep metrics for four classifiers: SLAMSS with activity, HRM, and HRSD inputs (SLAMSS-Act-HR), LSTM with activity, HRM, and HRSD inputs (LSTM-Act-HR), SLAMSS with HRM and HRSD inputs (SLAMSS-HR), and SLAMSS with an activity input (SLAMSS-Act). The orange dotted line corresponds to the PSG (assumed ground truth) value of each metric.

Clinical sleep metrics for the MrOS participants are shown in Fig 5, and a comparison of MAE for clinical sleep metrics is reported in Table 6. SLAMSS-Act-HR outperforms LSTM-Act-HR in all clinical sleep metrics except NREM time. In addition, compared to LSTM-Act-HR, all SLAMSS variants exhibit lower error values for sleep onset latency and sleep fragmentation. The most notable difference in the MrOS cohort between the classifiers is in REM sleep time estimation. While LSTM-Act-HR, SLAMSS-HR, and SLAMSS-Act overestimate REM time by 87%, 89%, and 272% respectively, the corresponding number is only 42% for SLAMSS-Act-HR. NREM sleep estimation is comparable between the top contenders—SLAMSS-Act-HR, LSTM-Act-HR, and SLAMSS-HR. But the high performance of SLAMSS-Act-HR at wake epoch classification contributes to a clear improvement in NREM fraction using this approach (76% vs. 84% for the PSG ground truth, 68% for LSTM-Act-HR, 66% for SLAMSS-HR, and 34% for SLAMSS-Act).

**Table 6:** MrOS three-class sleep staging. Comparison of MAE for clinical sleep metrics for four classifiers against PSG: SLAMSS with activity, HRM, and HRSD inputs (SLAMSS-Act-HR), LSTM with activity, HRM, and HRSD inputs (LSTM-Act-HR), SLAMSS with HRM and HRSD inputs (SLAMSS-HR), and SLAMSS with an activity input (SLAMSS-Act). MAE values are provided in the format: mean (s.d.).

Metric	SLAMSS-Act-HR	LSTM-Act-HR	SLAMSS-HR	SLAMSS-Act
**Sleep efficiency**	0.07 (0.06)	0.11 (0.10)	0.16 (0.17)	0.10 (0.09)
**Sleep onset latency (min.)**	44.07 (79.58)	67.89 (103.80)	59.25 (87.88)	56.42 (100.53)
**Sleep fragmentation**	0.33 (0.54)	0.43 (0.59)	0.41 (0.57)	0.41 (0.62)
**Sleep transition index**	0.05 (0.11)	0.07 (0.11)	0.07 (0.14)	0.06 (0.11)
**NREM time (hrs.)**	0.70 (0.72)	0.69 (0.75)	1.03 (1.01)	1.96 (1.37)
**REM time (hrs.)**	0.54 (0.63)	0.84 (0.84)	0.89 (0.75)	2.21 (1.49)
**Total sleep time (hrs.)**	0.46 (0.46)	0.74 (0.65)	1.11 (1.24)	0.73 (0.78)
**NREM fraction**	0.11 (0.11)	0.21 (0.21)	0.19 (0.16)	0.51 (0.21)
**REM fraction**	0.11 (0.11)	0.21 (0.21)	0.19 (0.16)	0.51 (0.21)

### Three-class sleep staging using smartwatch data

To assess the broad utility of the model across devices and specifically test its accuracy for smartwatch data, we conducted an independent validation of our model on a smartwatch dataset made available by Walch et al. [46]. This dataset consists of PPG-derived heart rate and raw accelerometry data obtained from the Apple Watch. The data size of 31 subjects by itself is small for training the parameter-heavy SLAMSS model. So, alongside attempting to directly train this model using the Apple Watch dataset, we also pursued a transfer learning approach using MESA, wherein a MESA-pretrained model’s entire structure was fine-tuned using the Apple Watch dataset by reducing the value of the learning rate from 0.00015 to 0.00001. The reason for excluding MrOS in pretraining is the fact that the MrOS dataset included only male subjects and its activity was measured per minute rather than per 15 s, which could potentially compromise the consistency of the Apple Watch data and pretraining cohort. We set aside 21 data samples for training and fine-tuning and 10 for independent testing. In addition, because the Apple Watch dataset is small, we performed 5-fold cross-validation without pretraining using SLAMSS.

**Fig 6:**
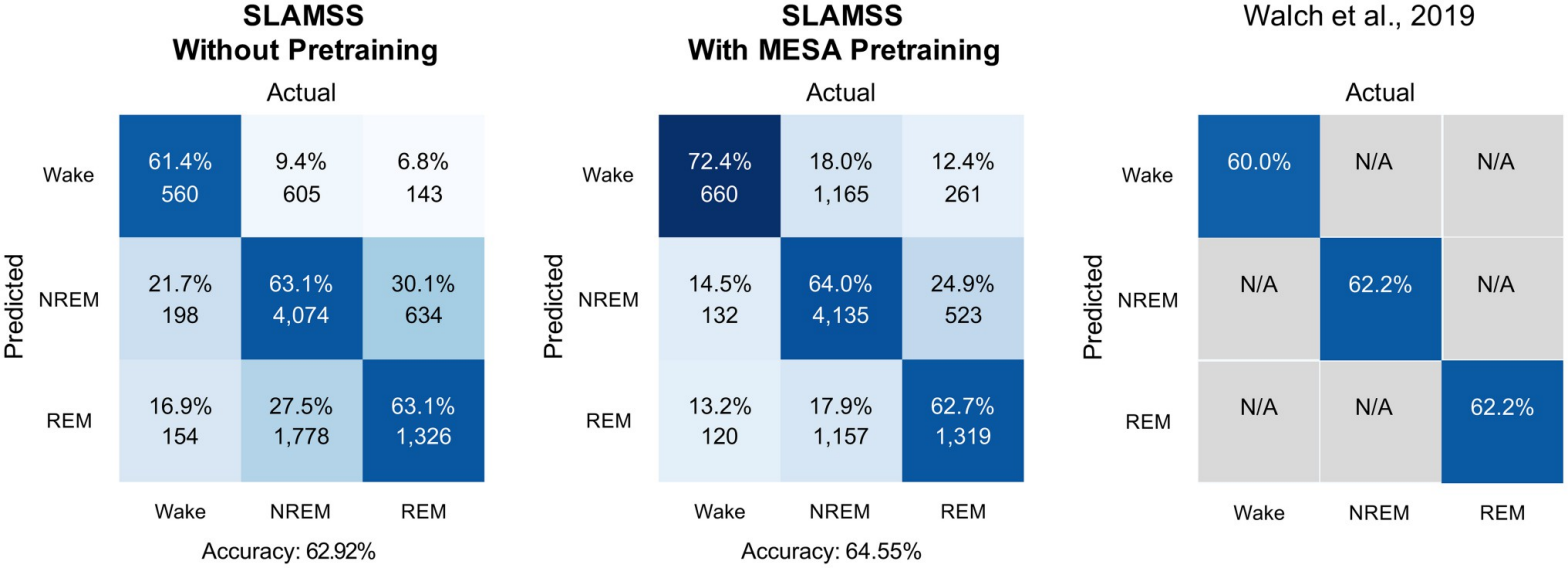
Confusion matrices for the SLAMSS model without MESA pretraining (i.e., direct training) and with MESA pretraining (i.e., transfer learning) with activity, HRM, and HRSD inputs for the Apple Watch dataset. The corresponding accuracies reported in [46] are provided for reference.

Confusion matrices for the direct training (i.e., no MESA pretraining) and transfer learning (i.e., with MESA pretraining) are shown in Fig 6. While the SLAMSS network has a higher “model capacity” and more degrees of freedom for learning, training it on smartwatch data without any pretraining results in only slightly higher accuracy compared to the 4-layer fully-connected neural network classifier described in [46]. This minor improvement is not unexpected, given that a higher model capacity may lead to overfitting when trained on a smaller dataset. In comparison, the MESA-pretrained model led to a more prominent boost in model performance, especially in the wake category. The cross-validation experiments for the model without pretraining led to a mean overall accuracy of 0.61 with a standard deviation of 0.04 for the model without pretraining. This mean value is similar to the result reported in Fig 6.

### Four-class sleep staging using the MESA dataset

**Fig 7:**
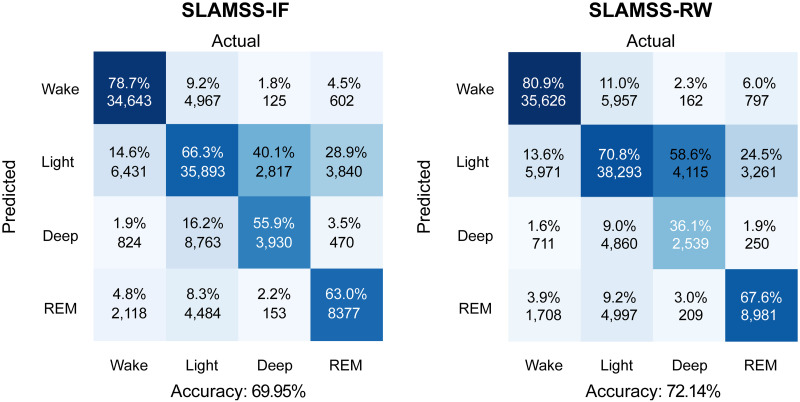
MESA four-class sleep staging. Confusion matrices for four-class sleep staging using SLAMSS with an inverse-frequency-weighted cross-entropy loss function (SLAMSS-IF) and SLAMSS with a real-world-weighted cross-entropy loss function (SLAMSS-RW). It should be noted that, for four-class staging, category assignment by random chance would lead to a value of 25% for the diagonal elements of these matrices.

Given the promising performance of SLAMSS for three-class staging, we next attempted the more challenging four-class staging task. We trained and tested the SLAMSS network to perform four-class sleep staging on the MESA cohort. The training and validation dataset sizes were the same as those used for three-class staging. We report in Fig 7 the four-class confusion matrix for SLAMSS trained using a cross-entropy loss function with IF weighting, the same loss function that was used for three-class staging. This model correctly identified 78.7% of wake epochs, 66.3% of light sleep (N1+N2) epochs, 55.9% of deep sleep (N3) epochs, and 63.0% of REM sleep epochs. With a MESA data distribution consisting of 33% wake epochs, 47% light sleep (N1+N2) epochs, 9% deep sleep (N3) epochs, and 11% REM sleep epochs, there is a severe underrepresentation of the deep sleep category. Although REM epochs constitute 11% of the total epochs, our coarse feature set appears to be more sensitive to the difference between REM and NREM and less discriminative of NREM sub-classes. IF weighting of the loss function mitigates this effect to some extent, as reflected by the 55.9% accuracy of the deep sleep stage (random chance would lead to a 25% accuracy for the four-class problem). This result also uses the clock time (raw value from the actigraphy device) as an input in addition to activity, HRM, and HRSD. In addition, we also incorporate the clock time for three-class staging, and the result is shown in S3 Fig. This inclusion was based on the insight that long deep sleep cycles occur in the first half of the night and become rarer as the night progresses. Thus, a temporal correlation exists between clock time and deep sleep, one that may be utilized by the network to differentiate between sleep stages. In comparison with our results, prior work by others on four-class sleep staging led to deep sleep accuracies of 4% [59] and 30.4% [60]. Relative to these past efforts (both of which used handcrafted ECG features), our deep sleep accuracy is a significant improvement. However, it should be noted that, for the IF-weighted loss function, 16.2% of light sleep epochs got misclassified as deep sleep. Light sleep, being the majority class, corresponds to a high number (8, 763) of false positive epochs in the deep sleep category compared to 3, 930 true positives. In other words, despite the reasonable deep sleep classification accuracy, this method ends up greatly overestimating the deep sleep class.

To mitigate class imbalance and further improve the classifier performance for the minority class (deep sleep), we used an RW-weighted loss function. A comparison of SLAMSS four-class sleep staging performance with the IF-weighted cross-entropy loss (henceforth referred to as SLAMSS-IF) and with the RW-weighted cross-entropy loss (henceforth referred to as SLAMSS-RW) is shown in Fig 7. With SLAMSS-RW, only 4, 860 light sleep epochs get misclassified as deep sleep compared to the much higher corresponding number of 8, 763 for SLAMSS-IF. SLAMSS-RW classifies 2, 539 actual deep sleep epochs as deep sleep compared to the number 3, 930 for SLAMSS-IF, leading to a drop in deep % accuracy. One should note, however, that while overall % accuracy is important, it does not offer the full picture. As shown in Table 7, the overall performance of SLAMSS-RW is comparable to (or perhaps slightly better than) SLAMSS-IF. A comparison of MAE for clinical sleep metrics against PSG is reported in Table 8. Light sleep time and deep sleep time results based on SLAMSS-RW outperform corresponding results from SLAMSS-IF by a large margin. The drop in the diagonal term of the confusion matrix accounts for the slightly lower sensitivity (64%) of SLAMSS-RW (compared to 66% for SLAMSS-IF). However, SLAMSS-RW has an improvement over SLAMSS-IF for the three metrics: 1% improvement in specificity, 1% in the weighted F1 score, and 2% in the MCC.

**Table 7:** MESA four-class sleep staging. Comparison of classifier performance metrics for four-class sleep staging using SLAMSS with an inverse-frequency-weighted cross-entropy loss function (SLAMSS-IF) and SLAMSS with a real-world-weighted cross-entropy loss function (SLAMSS-RW). PSG is used as the ground truth for the computation of all metrics. Subject-wise values are reported as mean(s.d.).

Metric	SLAMSS-IF	SLAMSS-RW
**Overall Accuracy**	0.70	0.72
**Sensitivity/Recall**	0.66	0.64
**Specificity**	0.89	0.90
**Precision**	0.61	0.61
**Weighted F1 score**	0.72	0.73
**Subject-wise**	0.72 (0.01)	0.73 (0.01)
**Weighted MCC**	0.56	0.58
**Subject-wise**	0.57 (0.02)	0.58 (0.02)

**Table 8:** MESA four-class sleep staging. Comparison of MAE for clinical sleep metrics for four-class sleep staging using SLAMSS with an inverse-frequency-weighted cross-entropy loss function (SLAMSS-IF) and SLAMSS with a real-world-weighted cross-entropy loss function (SLAMSS-RW) against PSG. MAE values are provided in the format: mean (s.d.).

Metric	SLAMSS-IF	SLAMSS-RW
**Sleep efficiency**	0.08 (0.07)	0.07 (0.08)
**Sleep onset latency (min.)**	28.57 (47.85)	24.46 (40.43)
**Sleep fragmentation**	0.19 (0.30)	0.19 (0.26)
**Sleep transition index**	0.04 (0.03)	0.04 (0.03)
**Light time (hrs.)**	0.98 (0.70)	0.88 (0.73)
**Deep time (hrs.)**	0.83 (0.75)	0.63 (0.65)
**REM time (hrs.)**	0.50 (0.38)	0.52 (0.51)
**Total sleep time (hrs.)**	0.77 (0.68)	0.76 (0.82)
**Light fraction**	0.13 (0.09)	0.12 (0.10)
**Deep fraction**	0.12 (0.10)	0.10 (0.09)
**REM fraction**	0.07 (0.06)	0.08 (0.07)

**Fig 8:**
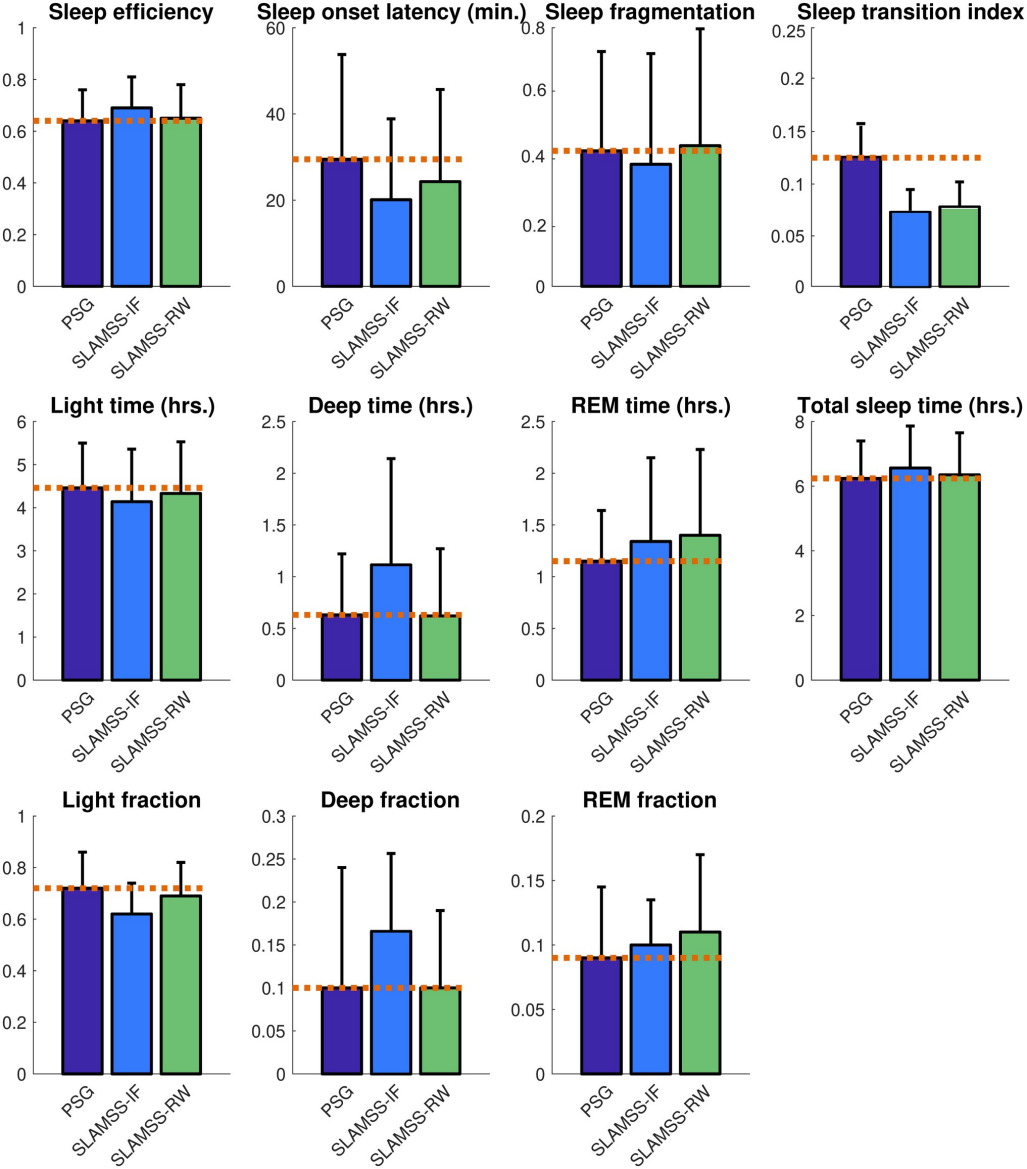
MESA four-class sleep staging. Comparison of clinical sleep metrics for four-class sleep staging using SLAMSS with an inverse-frequency-weighted cross-entropy loss function (SLAMSS-IF) and SLAMSS with a real-world-weighted cross-entropy loss function (SLAMSS-RW). The orange dotted line corresponds to the PSG (assumed ground truth) value of each metric.

The real benefit of SLAMSS-RW over SLAMSS-IF is evident when we examine clinical sleep metrics. As shown in Fig 8, while, for a number of these metrics, the two models have comparable performance, there is a striking difference in how the two methods quantify the two deep sleep metrics: deep sleep time and deep sleep fraction. SLAMSS-RW leads to highly accurate average values for both metrics: deep sleep time 0.61 hrs. (ground truth 0.60 hrs. from PSG) and deep sleep fraction 0.09 (ground truth 0.09 from PSG). The corresponding numbers for SLAMSS-IF are 1.16 hrs. (93% overestimation in the deep sleep time) and 0.17 (89% overestimation in the deep sleep fraction).

### Four-class sleep staging using the MrOS dataset

**Fig 9:**
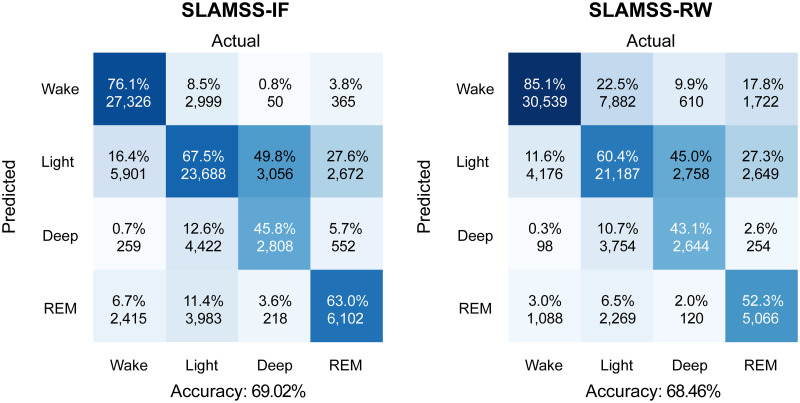
MrOS four-class sleep staging. Confusion matrices for four-class sleep staging using SLAMSS with an inverse-frequency-weighted cross-entropy loss function (SLAMSS-IF) and SLAMSS with a real-world-weighted cross-entropy loss function (SLAMSS-RW). It should be noted that, for four-class staging, category assignment by random chance would lead to a value of 25% for the diagonal elements of these matrices.

We compared the performance of SLAMSS-IF and SLAMSS-RW in the MrOS cohort. In this cohort, the data distribution consists of 45% wake epochs, 38% light sleep (N1+N2) epochs, 7% deep sleep (N3) epochs, and 10% REM sleep epochs. Both the deep and REM categories are underrepresented. As shown in Fig 9, SLAMSS-IF correctly identified 76.1% of wake epochs, 67.5% of light sleep (N1+N2) epochs, 45.8% of deep sleep (N3) epochs, and 63.0% of REM sleep epochs. In comparison, SLAMSS-RW correctly identified 85.1% of wake epochs, 60.4% of light sleep (N1+N2) epochs, 43.1% of deep sleep (N3) epochs, and 52.3% of REM sleep epochs. Relative to SLAMSS-IF, SLAMSS-RW led to drastic reductions in the off-diagonal terms in the confusion matrices under the deep and REM sleep categories. The two methods were roughly comparable in terms of standard metrics for assessing classifier performance listed in Table 9.

**Table 9:** MrOS four-class sleep staging. Comparison of classifier performance metrics for four-class sleep staging using SLAMSS with an inverse-frequency-weighted cross-entropy loss function (SLAMSS-IF) and SLAMSS with a real-world-weighted cross-entropy loss function (SLAMSS-RW). PSG is used as the ground truth for the computation of all metrics. Subject-wise values are reported as mean(s.d.).

Metric	SLAMSS-IF	SLAMSS-RW
**Overall Accuracy**	0.69	0.68
**Sensitivity/Recall**	0.63	0.60
**Specificity**	0.89	0.88
**Precision**	0.60	0.61
**Weighted F1 score**	0.69	0.68
**Subject-wise**	0.69 (0.01)	0.69 (0.01)
**Weighted MCC**	0.56	0.52
**Subject-wise**	0.56 (0.01)	0.54 (0.02)

**Fig 10:**
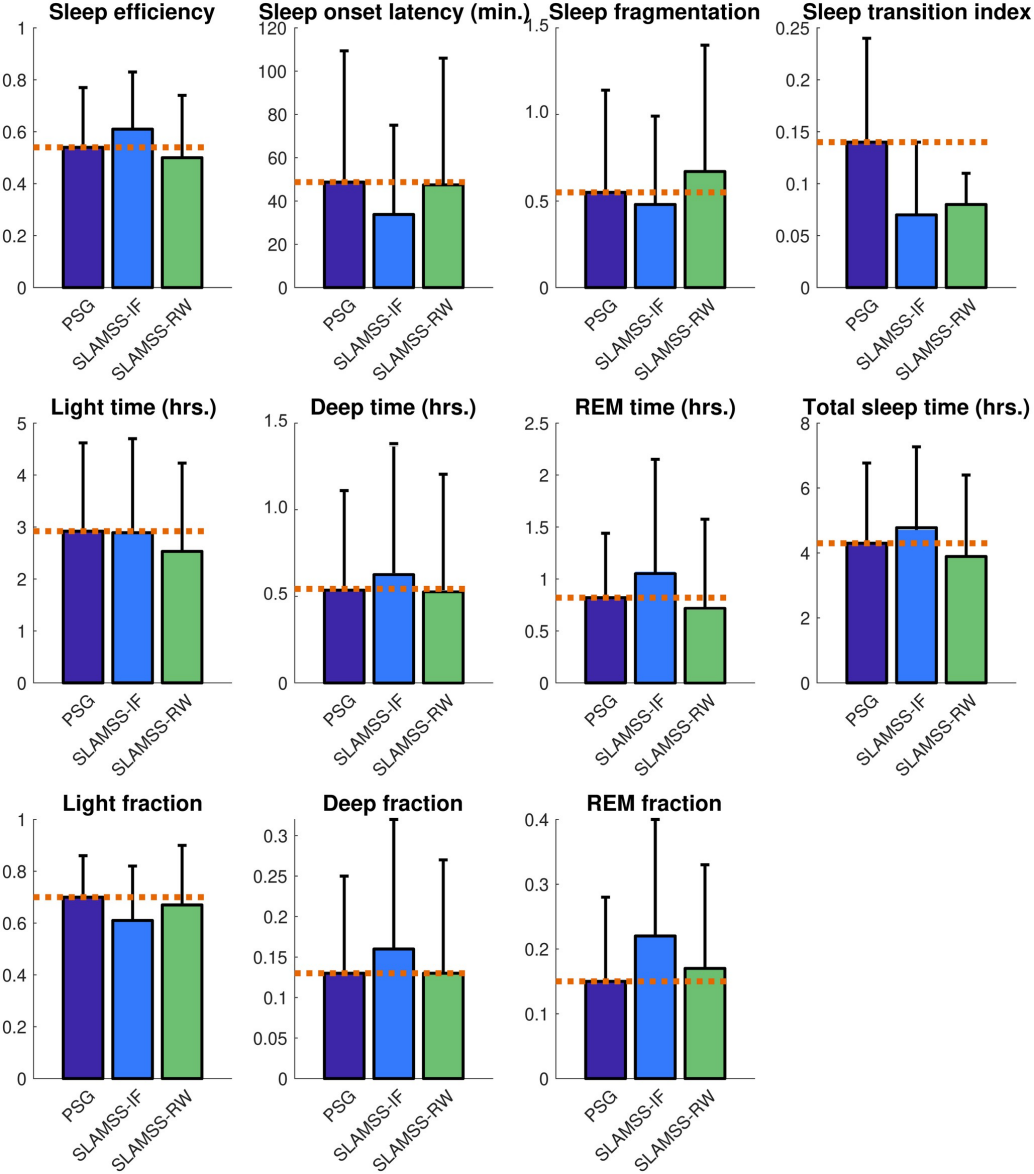
MrOS four-class sleep staging. Comparison of clinical sleep metrics for four-class sleep staging using SLAMSS with an inverse-frequency-weighted cross-entropy loss function (SLAMSS-IF) and SLAMSS with a real-world-weighted cross-entropy loss function (SLAMSS-RW). The orange dotted line corresponds to the PSG (assumed ground truth) value of each metric.

A comparison of clinical sleep metrics obtained from SLAMSS-RW over SLAMSS-IF with PSG-based metrics as reference is shown in Fig 10, and a comparison of MAE for clinical sleep metrics is reported in Table 10. In terms of deep time, SLAMSS-RW performs better than SLAMSS-IF. SLAMSS-RW mitigates overestimation of both deep and REM sleep time relative to SLAMSS-IF. As in MESA, SLAMSS-RW is especially accurate at measuring both deep sleep time and REM sleep time: deep sleep time 0.53 hrs. (ground truth 0.55 hrs. from PSG) and REM sleep time 0.71 hrs. (ground truth 0.80 hrs. from PSG). The corresponding numbers for SLAMSS-IF are 0.64 hrs. (16% overestimation in the deep sleep time) and 1.05 hrs. (31% overestimation in the REM sleep time). Similar improvements were also observed in the deep and REM sleep fractions.

**Table 10:** MrOS four-class sleep staging. Comparison of MAE for clinical sleep metrics for four-class sleep staging using SLAMSS with an inverse-frequency-weighted cross-entropy loss function (SLAMSS-IF) and SLAMSS with a real-world-weighted cross-entropy loss function (SLAMSS-RW) against PSG. MAE values are provided in the format: mean (s.d.).

Metric	SLAMSS-IF	SLAMSS-RW
**Sleep efficiency**	0.08 (0.08)	0.11 (0.13)
**Sleep onset latency (min.)**	24.90 (44.14)	27.09 (42.55)
**Sleep fragmentation**	0.30 (0.47)	0.46 (0.74)
**Sleep transition index**	0.09 (0.11)	0.08 (0.11)
**Light time (hrs.)**	0.74 (0.65)	0.82 (0.88)
**Deep time (hrs.)**	0.50 (0.68)	0.40 (0.45)
**REM time (hrs.)**	0.45 (0.53)	0.38 (0.38)
**Total sleep time (hrs.)**	0.58 (0.54)	0.83 (1.17)
**Light fraction**	0.16 (0.14)	0.15 (0.18)
**Deep fraction**	0.12 (0.12)	0.01 (0.11)
**REM fraction**	0.09 (0.10)	0.08 (0.08)

## Discussion

Sleep staging outcomes from MESA were successfully replicated in the MrOS cohorts. The two independent study populations have very different demographics: Compared to MESA, the MrOS cohort is older, all male, and predominately white. Whereas the MESA actigraphy device outputs activity counts at a 30-s-epoch temporal resolution, the MrOS device provides coarser activity reads with a 60-s-epoch temporal resolution. The small differences in accuracies between the two cohorts are therefore attributable not only to demographic differences but also to the use of different actigraphy devices.

A key contribution of this paper is the accurate estimation of NREM sleep time (in three-class staging) and N3/deep/slow-wave sleep time (in four-class staging). This capability allows us to go beyond the simple sleep-wake scoring functionality offered by current smartwatches. A number of serious disorders are heralded by or associated with a reduction of slow-wave sleep. For example, there is increasing evidence that sleep disturbances, including features such as reduced NREM slow-wave activity at 1–2 Hz and disruption of NREM slow oscillation-spindle phase coupling, might be the earliest observable symptoms of Alzheimer’s disease (AD) and tend to surface before or soon after the diagnosis of cognitive impairment [64, 65]. Routine monitoring of N3 sleep time has the potential to identify subjects at risk of disorders such as AD.

While overall accuracy is the most popular benchmark for assessing the performance of a machine learning model, this metric has some limitations. Firstly, it fails to capture the effect of all terms of the confusion matrix. Secondly, being a frequency-weighted average measure across different classes, it can be especially misleading for imbalanced multi-class problems. This is due to the fact that the mean value tends to not be reflective of the performance of the underrepresented class when there is class imbalance. We, therefore, report full confusion matrices for individual cases and supplement them with the F1 score and MCC as scalar performance measures. The MCC, in particular, is regarded as a balanced measure that is useful even where the class imbalance is severe. We refrain from the use of Cohen’s *κ* in light of recent literature that indicates its unsuitability for imbalanced multi-class classification problems [66]. In severely imbalanced scenarios, a worse classifier (i.e., one with more false positives and true negatives) could have a higher *κ* value. This means that this performance measure could significantly diverge from the MCC, a measure that accounts for all elements of the confusion matrix.

A key weakness of this work is the fact that the heart rate data for MESA and MrOS is derived from lab-grade devices. However, we would like to emphasize that, although the heart rate measures for both MESA and MrOS were obtained from lab-grade ECG, we coarsened the temporal resolution to 30 s, a figure that PPG-based heart rate tracking devices can easily outperform, ensuring our method’s broader applicability to smartphone-derived heart rate measures. As our future work, therefore, we plan to train and test SLAMSS on data from wristworn devices that can generate both activity and heart rate measures.

Deep sleep classification is generally recognized to be a challenging task. Class imbalance, i.e., the underrepresentation of deep sleep, is a major reason for this. Additionally, thresholds for deep/light feature discrimination could be dependent on demographic variables, such as age and sex, further compounding the challenge [67]. Moreover, cardiac and actigraphic values lie on a continuum for light and deep sleep, which are both associated with the relaxed movement of muscles and slowing of the heart rate, making binarization difficult. It is well-known that deep sleep occurs for longer uninterrupted periods in the first half of the night. Our analysis showed that deep sleep, at a population level, largely occurred between 9 pm and 11 pm in the training dataset. To exploit this information, we utilized the raw clock time, which boosted deep sleep staging accuracy as shown in S4 Fig. This feature is ubiquitous among nearly all sleep-sensing devices and could be an inexpensive feature to help discern deep sleep.

Whereas three-class sleep staging suffers from some class imbalance with the underrepresentation of REM (11% REM, 33% wake, and 56% NREM epochs in the MESA dataset; 10% REM, 45% wake, and 45% NREM epochs in the MrOS dataset), IF weighting seems sufficient for addressing this imbalance. The relative ease of three-stage classification could also be due to the fact that activity and heart rate features are sufficiently discriminative for the three classes. REM sleep has cardiac measures similar to wakefulness and yet low activity counts (due to muscle atonia) [68, 69], making it discernible from both wake and NREM categories from coarse activity and heart rate measures. Thus, SLAMSS, which relies on coarse activity and heart rate inputs, demonstrates robust REM/NREM classification despite the underrepresentation of REM. Notably, while modified clock-based features have been reported by others to facilitate three-class staging [46], we observed no similar corresponding gain for the three-class case with our raw clock-time input, as shown in S3 Fig.

While all SLAMSS variants outperformed LSTM at estimating sleep onset latency, this metric’s deviation from the ground truth remained substantial. We note here that we have used the most stringent definition for this metric, which is based on the first detected period of three consecutive sleep epochs. This definition leads to reliable measures with an information-rich modality such as the EEG. But severe underestimation of sleep onset latency has been reported when calculated from actigraphy.

In current literature, the sleep fragmentation metric is used to capture the discontinuity of sleep. Sleep fragmentation depends on the binary classification of sleep and wake stages. For a more nuanced picture of sleep discontinuities, we defined a new metric known as the sleep transition index. Unlike sleep fragmentation, this index captures shifts between the individual stages of sleep and depends on multi-class sleep staging. It computes, for the post-sleep-onset period, the ratio of the time spent in sleep transitions to the total sleep time. It is reflective of the incidence of sleep stage shifts and captures the underlying sleep dynamics, particularly the instability of sleep stages across a person’s sleep span. For future work, we will investigate new transition metrics for individual sleep stages, e.g., a deep sleep transition index or a REM sleep transition index. Such stage-specific transition indices would be useful for detecting the onset of disorders that manifest as changes in specific sleep stages.

Overall, SLAMSS represents a significant advance toward a fully automated, clinically relevant, and objective approach for computing sleep stages from heart rate and movement data derived from smartwatches or other wrist wearables. It could enable long-term and population-scale sleep assessment and is therefore clinically promising.

## Conclusion

We have demonstrated the ability of the SLAMSS platform to perform multi-class (i.e., three- and four-class) sleep staging based on feature-poor actigraphic and cardiac inputs that can be easily obtained from a wide variety of smartwatch devices. The majority of AI-based sleep staging techniques thus far have focused on feature-rich EEG and ECG datasets [27, 30, 32], neither of which are available from wrist-based metrics generated in real time by consumer smartwatches. While some smartwatches offer a single-lead ECG functionality, the data acquisition requires the active engagement of the user and is not suited for continuous monitoring. By focusing on input modalities that are commonly available, we ensure the scalability and broad utility of our method. Traditionally, EEG and ECG-based sleep scoring has relied on extensive handcrafting of time-series features. Our reliance on a representation learning paradigm, i.e., a deep neural network that simultaneously optimizes the feature sets and parameters, obviates the need for manual feature selection. The SLAMSS architecture proposed here uses front-end convolutional layers but ultimately relies on an attention-guided Seq2Seq network that is able to learn temporal correlations. Whereas standalone CNNs have been successfully used for sleep staging from ECG-based instantaneous heart rate data with high temporal resolution in [32], we have found that in a setting such as ours where the temporal resolution is low (i.e., 30-s long epochs), CNN features alone are not sufficiently informative for accurate classification.

## Supporting information

S1 Table Definitions of metrics for assessing classifier performance. (TIF)

S2 Table Definitions of clinical sleep metrics. (TIF)

S1 Fig RWL false-negative and false-positive weight matrices (*W*_*fn*_ and *W*_*fp*_ respectively). *w* is the ratio of wake epochs to all epochs. *l* is the ratio of Light epochs to all epochs. *d* is the ratio of deep epochs to all epochs. *l* is the ratio of REM epochs to all epochs.
(TIF)

S2 Fig Confusion matrices for three-class sleep staging using SLAMSS-Act-HR (based on a standard IF-weighted cross-entropy loss function) with epoch lengths of 9, 12, and 15. (TIF)

S3 Fig Confusion matrices for three-class sleep staging using SLAMSS (based on a standard IF-weighted cross-entropy loss function) with activity, HRM, and HRSD inputs (SLAMSS-Act-HR), and SLAMSS with activity, HRM, HRSD, and raw clock time inputs (SLAMSS-Act-HR-Clock) with PSG being used as the ground truth. (TIF)

S4 Fig Confusion matrices for four-class sleep staging using SLAMSS-RW with activity, HRM, and HRSD inputs (SLAMSS-RW-Act-HR), and SLAMSS-RW with activity, HRM, HRSD, and raw clock time inputs (SLAMSS-RW-Act-HR-Clock) with PSG being used as the ground truth. (TIF)
