# Supplementary figures and images for: AI-Driven sleep staging from actigraphy and heart rate
Source: PLoS One. 2023 May 17;18(5):e0285703. doi: 10.1371/journal.pone.0285703 (PMC10191307; doi:10.1371/journal.pone.0285703)

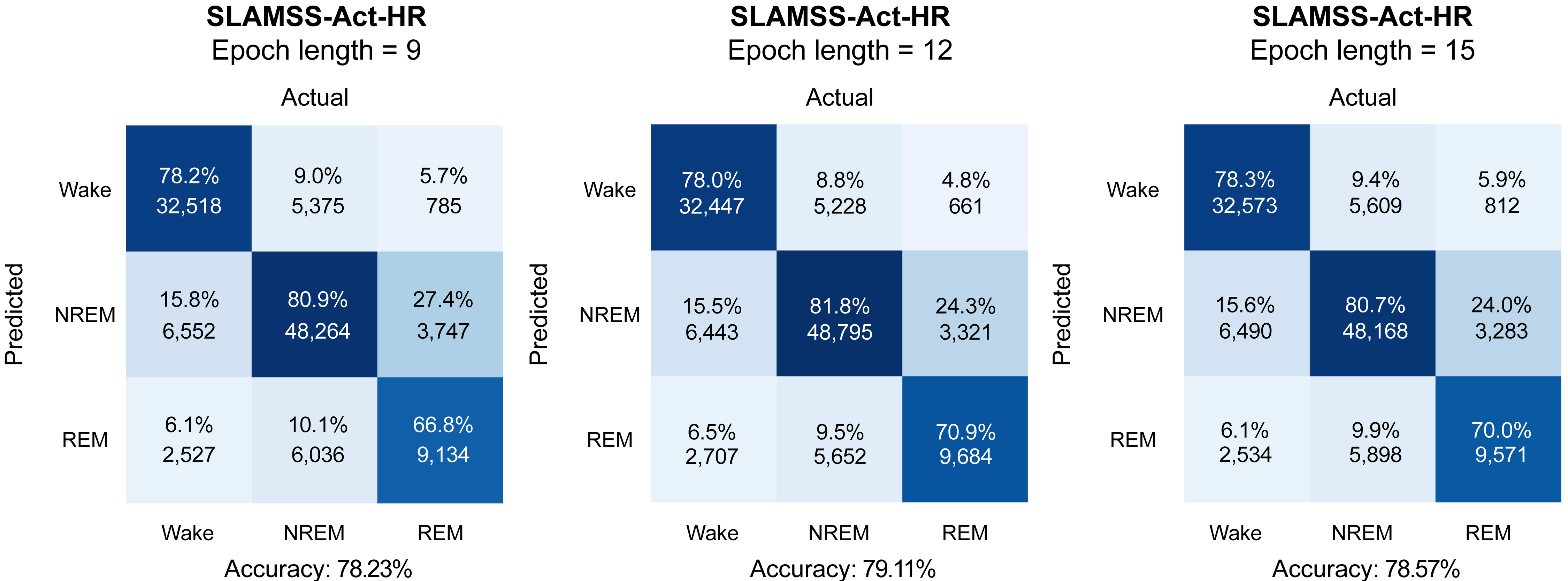

Supplement: S2 Fig — (TIF) [file pone.0285703.s004.tif]

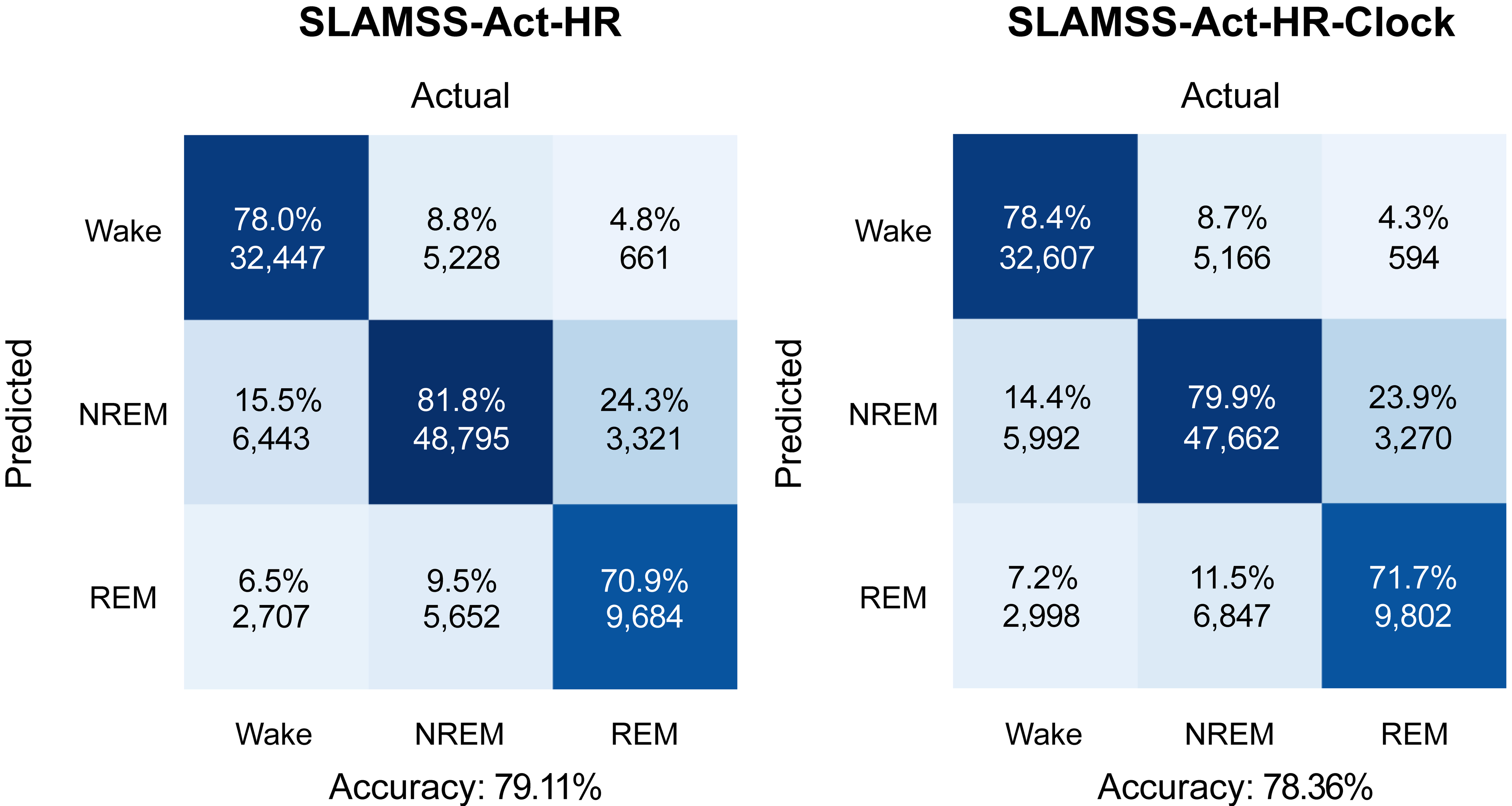

Supplement: S3 Fig — (TIF) [file pone.0285703.s005.tif]

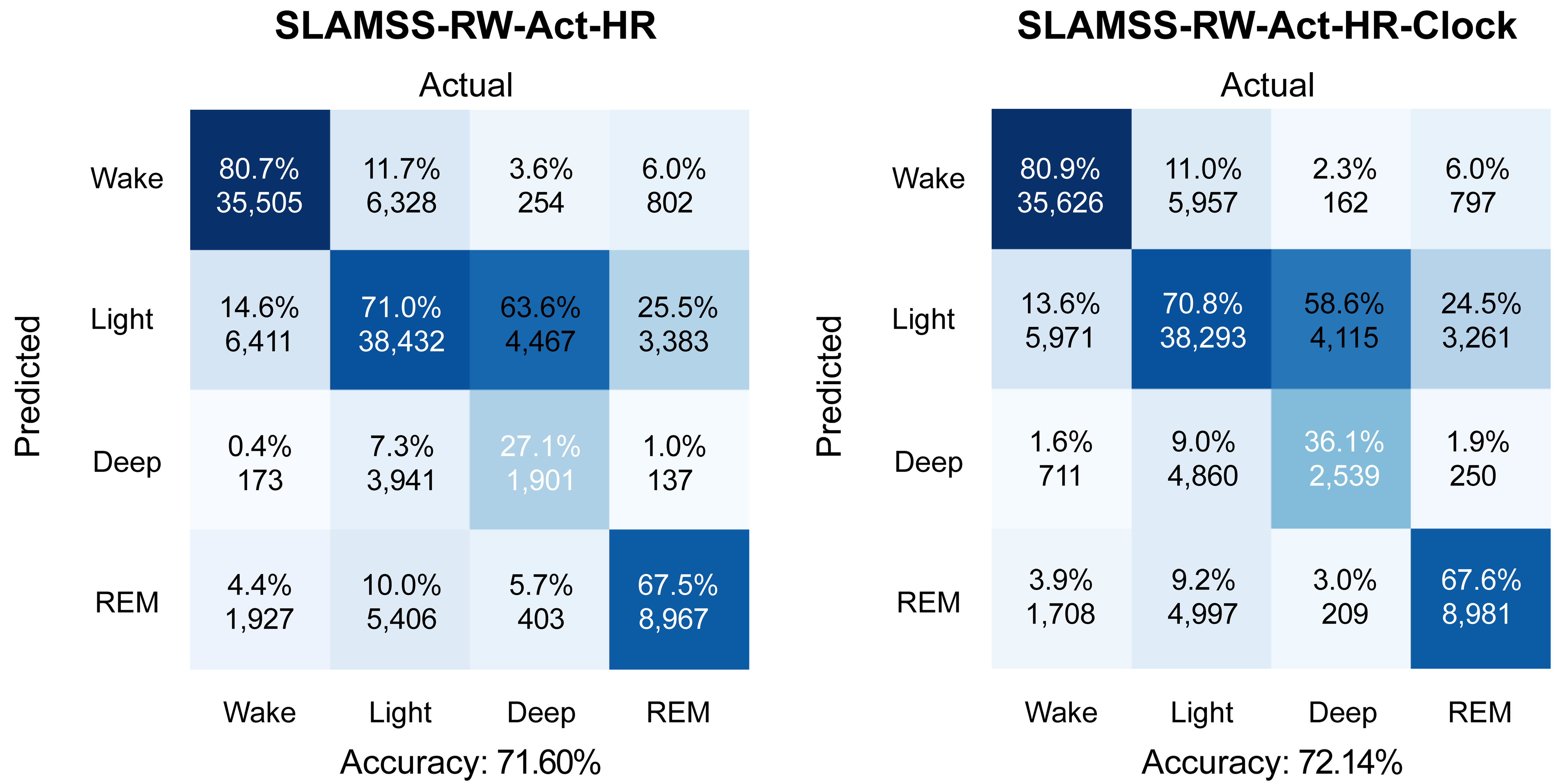

Supplement: S4 Fig — (TIF) [file pone.0285703.s006.tif]
